# Evaluation of a Treadmill-Based Submaximal Fitness Test in Pugs, and Collecting Breed-Specific Information on Brachycephalic Obstructive Airway Syndrome

**DOI:** 10.3390/ani12121585

**Published:** 2022-06-19

**Authors:** Rebekka Mach, Pia S. Wiegel, Jan-Peter Bach, Martin Beyerbach, Lothar Kreienbrock, Ingo Nolte

**Affiliations:** 1 Clinic for Small Animals, University of Veterinary Medicine Hannover, Foundation, 30559 Hannover, Germany; pia.saskia.wiegel@tiho-hannover.de (P.S.W.); jpbach81@gmail.com (J.-P.B.); 2 Institute for Biometry, Epidemiology and Information Processing, University of Veterinary Medicine Hannover, Foundation, 30559 Hannover, Germany; martin.beyerbach@tiho-hannover.de (M.B.); lothar.kreienbrock@tiho-hannover.de (L.K.)

**Keywords:** BOAS, brachycephaly, dogs, exercise testing

## Abstract

**Simple Summary:**

In the present study, a submaximal fitness test on a treadmill was evaluated to assess its feasibility under standardised conditions. Moreover, its effectiveness in identifying pugs with clinical signs of brachycephalic obstructive airway syndrome was examined. It was apparent that respiratory symptoms can be exposed during the fitness test, and intensified with an increased duration of exercise. This method of testing improves the clinical evaluation of the dogs and helps identify restrictions due to brachycephalic obstructive airway syndrome. Since most of the dogs could be familiarised with the treadmill, it may be a feasible option for performing fitness tests in brachycephalic dogs. Major benefits, therefore, are that each dog can be closely monitored during the entire process, and that the examination can be conducted under standardised conditions.

**Abstract:**

Despite efforts of veterinarians and breeders, brachycephalic obstructive airway syndrome (BOAS) is still a common problem in pugs, underlining the need for objective tests to identify and prevent breeding with affected dogs. In the current study, a submaximal, treadmill-based fitness test was evaluated as a tool to identify signs of airway obstruction not recognisable under rest conditions. In addition to this, different body conformation and measurements were assessed regarding their association with BOAS. A total of 62 pugs and 10 mesocephalic dogs trotted with an individual comfort speed on a treadmill for 15 min. Before and during the examination, dogs were examined for signs of respiratory distress, and a functional BOAS grading was applied. The influence of body conformation on BOAS grading was tested in a univariable and multivariable logistic regression model. During exercise, more respiratory noises were observed, and existing respiratory noises became more apparent in comparison to when at rest. In the multivariable logistic regression model, no factor had a statistically significant influence on BOAS classification. Submaximal fitness testing helped to identify signs of respiratory distress not apparent under resting conditions, and could be a valuable addition for identifying dogs with BOAS. Performing testing on a treadmill facilitates continuous observation of the patients, and enables standardisation of the test regarding the test environment, as well as provides an uninterrupted, steady workload.

## 1. Introduction

Brachycephaly is the congenital shortening of the skull and muzzle as a result of breeding [1,2,3,4]. Brachycephalic dogs show various characteristics of young animals and humans, such as a large head, and deep- and wide-set eyes, which give them an adorable appearance and make them instinctively attractive to humans [5]. Several studies have shown that breeding for a specific appearance such as brachycephaly is associated with a predisposition to certain diseases [6,7,8]. One of the diseases associated with shorter muzzles is brachycephalic obstructive airway syndrome (BOAS), which occurs frequently in brachycephalic dogs but is not present in all of them [9,10,11]. BOAS-affected dogs show a variety of possible symptoms, such as loud respiratory noises (RN); exercise and heat intolerance; gastrointestinal problems; and respiratory distress, including severe symptoms such as cyanosis and choking fits [11,12,13,14,15,16,17,18,19,20]. Symptoms of BOAS have long been reported in brachycephalic dogs, with the first description of surgical intervention reported in 1929 [21]. Despite extensive evidence of BOAS in the veterinary literature, brachycephalic dogs are still popular [22,23,24].

Studies on conformational risk factors for BOAS-affected dogs show a huge variation [9,25,26]. In one study, by Packer et al., the craniofacial ratio (CFR) was identified as having a strong impact on BOAS risk in all investigated breeds, including the pug [25]. In two other studies, however, the CFR showed only a weak association, while other risk factors, such as stenotic nostrils, had a higher impact on the risk of being BOAS-affected in pugs and French bulldogs [9,26]. In these three studies, the classification of BOAS-affected and unaffected dogs was performed with different methods.

An important factor in studies concerning BOAS is its negative functional impact on exercise capacity, which affects the daily life of the dogs [19,26]. Exercise tests have been proven to be effective in reflecting exercise capacity in humans [27,28,29], and a submaximal workload seems suitable to reflect the exercise level for daily physical activities [28,29,30]. Exercise tests have also been proven to be effective in identifying BOAS-affected animals, as BOAS-associated symptoms are more evident, and can be assessed more easily under exercise conditions [11,31]. A submaximal exercise test that is often used in humans, but the precise instructions of which are difficult to implement in dogs, is the six-minute walk test [28,30]. In comparison to human patients, ensuring a comparable level of motivation to walk is impossible in canine patients. This is problematic, because the amount of encouragement can make a difference of up to 30% in outcome measurements in human patients [28]. Additionally, a 1000-metre walking test was shown to be more effective for identifying BOAS-affected dogs than the six-minute walk test [11].

An exercise test with the aim of improving the health of brachycephalic dogs has been used for breeding approval in Germany since 2009 [32]. However, there is no standardised exercise protocol, the speed is not specified, and the dogs are not monitored throughout the test or the recovery phase [32]. This limitation in existing tests, in conjunction with the persistent existence of BOAS in Germany and worldwide, suggests the need for improved tests to identify and prevent breeding with affected dogs.

A standardised, submaximal exercise test on a treadmill has already been developed and tested in dogs with early mitral valve disease [33], and has been used to investigate the effect of medication in these patients [34,35]. These studies, and others, showed that it is possible to acclimatise untrained dogs to a treadmill [33,34,36,37,38]. In addition to this, the studies verified that fitness tests on treadmills provide reliable and reproducible results [33,36].

The aim of the present study was to evaluate an individual, submaximal fitness test (FT) on a treadmill under standardised conditions and to examine parameters, such as RN and breathing patterns, in the context of the specific workload. The results of the FT were also evaluated in relation to a functional BOAS classification of the pugs. Further examination of possible anatomical risk factors for BOAS in this study population were investigated with the aim of improving our knowledge of the disease. The FT was first performed on pugs as a representation of a brachycephalic breed.

## 2. Materials and Methods

### 2.1. Study Animals

The study was a prospective study, and was performed at the Clinic for Small Animals of the University of Veterinary Medicine, Foundation, Hannover, Germany, between July 2019 and August 2020. Privately owned pugs aged a minimum of two years were eligible for inclusion in the study. Dogs were acquired via social media, including a call to participate from the German Kennel Association (Verband für das deutsche Hundewesen, VDH), and some dogs were acquired from the database of the Clinic for Small Animals. Ten healthy mesocephalic dogs (five border terriers, five mongrels) of similar age and weight to the study population of pugs, were included as a control group (CG). Exclusion criteria were relevant systemic diseases apart from BOAS and/or previously performed upper airway surgery. Pugs from FCI (Fédération Cynologique Internationale)-registered and unregistered breeding associations were accepted for the study. In total, 62 pugs were included in this study.

Before starting the FT, the dogs’ identification data (chip number, date of birth, pedigree, etc.,) were recorded, and all dogs underwent physical examination. Owners filled in a questionnaire, including questions related to possible breathing problems, breathing sounds, or problems related to digestion (File S1).

### 2.2. Submaximal Fitness Test

The submaximal FT was performed on a motorised treadmill (“quasar”, h/p/cosmos sports and medical GmbH, Nussdorf-Traunstein, Germany). Conditions in the examination room were controlled by air conditioning: the temperature was kept between 20–24 °C and the humidity within a range of 30–60%. A blood sample was taken from each dog for analysis, and dogs had 15 min to acclimatise to the setting of the FT. This was followed by a 10-min familiarisation period, in which the dogs learned how to run on the treadmill, being motivated by the owners by verbal encouragement and food. One investigator secured the dog with a harness and was always able to stop the treadmill if necessary. An individual comfort speed between four and eight kilometres per hour (km/h) was identified for each dog. The comfort speed was considered to be reached when the dog trotted steadily without trying to stop or attempt to slow down or speed up. After the familiarisation period, there was a 15-min break before the actual FT started. During the FT, the dogs trotted for 15 min at their individual comfort speed on the treadmill, with a one-minute measurement break after trotting for five minutes. With the measurement breaks, the FT lasted 17 min in total. Heart rate (HR), rectal body temperature (T), and respiratory rate (RR) were documented at predetermined time points (Table 1). HR was monitored continuously using a Polar heart rate monitor (Polar FT7N and Polar H1, Polar Electro GmbH Deutschland, Büttelborn, Germany). Usage and reliability of the Polar system for dogs has been previously described [39]. To ensure a minimal workload, the HR had to rise by 40% compared to the resting value. To ensure that the load did not exceed the submaximal range, the FT was stopped when an HR of 220 beats per minute (bpm) was reached [40]. T was taken using a digital thermometer. After performing the FT, a 15-min recovery period followed. During this time, HR, T, and RR were documented at five-minute intervals. HR and RR were expected to return to baseline, with a tolerance of 10%, and T should decrease to the normal range of 38.0–39.2 °C [41]. If this was not the case, the values were re-evaluated every two minutes.

### 2.3. Assessment of Respiratory Noises and Breathing Patterns

RN and breathing patterns in the form of intensity of inspiratory effort and possible dyspnoea were assessed at rest and after 5, 10, and 15 min of exercise. At all times, the classification was performed in identical fashion (Table S1). We evaluated whether an RN was audible with or without a stethoscope. Audible noises were classified as intermittent or constant. The intensity was subjectively categorised into mild, moderate, or severe. In addition, the sounds were classified according to their assumed origin as either stertor (associated with vibrations of flaccid tissues in the upper portion of the airway, such as an elongated soft palate [15,17,42]), stridor (associated with constriction of rigid tissues, such as that caused by laryngeal narrowing [15,17,31]), or “not assignable”. The intensity of inspiratory effort was described as absent, mild (minimal additional use of the diaphragm), moderate (distinct use of diaphragm and accessory respiratory muscles), or severe (intensive use of the diaphragm and accessory respiratory muscles). The presence of dyspnoea was assessed as absent, mild (signs of discomfort), moderate (irregular breathing), or severe (irregular breathing with clear signs of discomfort).

### 2.4. Functional BOAS Grading

The functional grading system, originally designed and validated by Liu et al. in 2015, was modified to match the present study design [43]. With this grading system, the dogs were classified as having no (grade 0), mild (grade 1), moderate (grade 2), or severe (grade 3) signs of BOAS. BOAS grading was performed by evaluating whether RNs were audible with or without a stethoscope, assessing the inspiratory effort and signs of dyspnoea or cyanosis before and after 5, 10, and 15 min of exercise. For a comparison between functionally impaired and unimpaired dogs, the dogs were further divided into BOAS− (grades 0 and 1) and BOAS+ (grades 2 and 3), as previously described [43]. Dogs that could be classified at least with BOAS grade 2 according to their symptoms at rest, and thus did not walk on the treadmill, were also classified as BOAS+.

### 2.5. Assessment of Body Conformation and Measurements

The weight of all dogs was recorded, and body condition score (BCS) was assessed using a 1–9-point scale [44]. Stenosis of the nostrils was visually described as previously reported [9], and graded from open (0) to mild (1), moderate (2), or severe (3) stenosis. For further investigation on the influence of BCS and stenosis of the nostrils on BOAS grading, dogs were divided into groups of normal weight (BCS 4–5) and overweight (BCS 6 –9), and stenosis of the nostrils was summarised as existing (grades 2 and 3) and not existing (grades 0 and 1).

Body measurements were evaluated according to Sutter et al. [45]. A detailed description of the measurements performed in this study can be found in the supporting information (Table S2). Skull length, eye width, chest girth, and body length were measured with a 1 m soft tape measure. Muzzle length was measured with a 20 cm ruler. Additionally, the CFR, i.e., the ratio of muzzle length to skull length, was calculated.

### 2.6. Statistical Analysis

For statistical analyses, SAS 9.4 and SAS Enterprise Guide 7.1 (SAS Institute, Inc., Cary, NC, USA) were used. Visual assessment of the QQ plots, and the Shapiro–Wilk test, was used to test for normally distributed data within the groups. If values were normally distributed, a t-test for independent samples was used to compare values between pugs and the CG. If data were not normally distributed, the Wilcoxon two-sample test was applied. To examine differences in HR and T values between the CG and pugs, subdivided into the BOAS groups, Fisher’s LSD (least significant difference) test was applied. Baseline characteristics of these groups were compared using the Kruskal–Wallis test. Recovery times were also compared using the Kruskal–Wallis test, and for pairwise comparison, the Wilcoxon two-sample test was applied. To investigate the influence of body conformation and the additional measurements on the BOAS grading, univariable and multivariable logistic regression was performed, BOAS-affected being the outcome variable. The level of significance was set at *p* < 0.05 in all tests with no adjustment for multiple testing.

## 3. Results

### 3.1. Submaximal Exercise Test

In total, 72 dogs were included in this study, 62 of which were pugs. The baseline characteristics of the pugs and the CG are shown in Table 4. There was no statistically significant difference between the pugs and CG in terms of age and weight. Furthermore, the adjusted individual comfort speed was not statistically significantly different between the groups, allowing one to assume a comparable workload. Overall, the FT was easy to perform, and most dogs were willing to run on the treadmill. Eight pugs and two dogs in the CG could not be familiarised with it. Furthermore, the FT had to be stopped for two pugs. In the case of one of these pugs, the HR increased to over 220 bpm after four minutes of exercise, whereupon the FT was terminated. In addition, at this time, this dog produced a constant severe pharyngeal and laryngeal breathing sound audible without a stethoscope, moderately increased inspiratory effort, and mild dyspnoea. The other pug exhibited a constant severe pharyngeal breathing sound audible without a stethoscope, severely increased respiratory effort, and moderate dyspnoea after five minutes of exercise. Due to difficulties with breathing, the FT was stopped at this point. The measurements of HR, T, and RR of these two pugs at rest and until FT termination were included in the statistical analysis.

**Figure 1:**
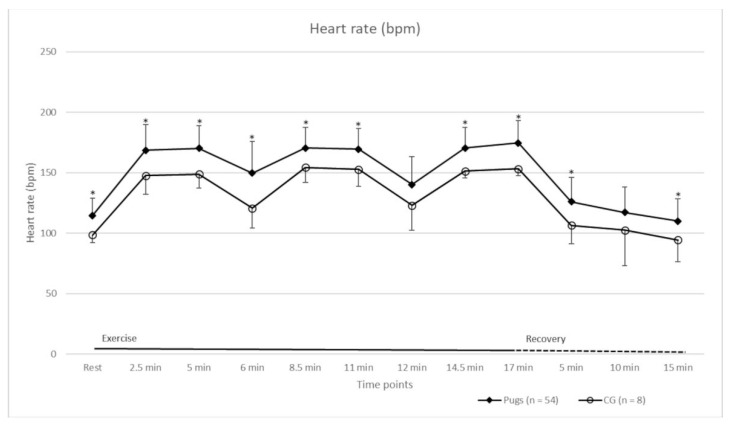
Mean heart rate (beats per minute, bpm) (± standard deviation) of pugs (*n* = 54) and control group (CG) (*n* = 8) at rest, during exercise, and throughout recovery period. Statistically significant differences between the groups at the measurement time points are indicated by an asterisk (*).

**Figure 2:**
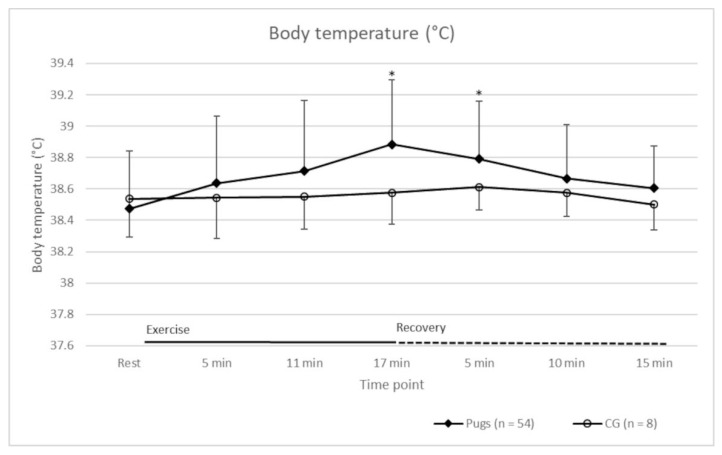
Mean body temperature (°C) (± standard deviation) of the pugs (*n* = 54) and the control group (CG) (*n* = 8) at rest, during exercise, and throughout recovery period. Statistically significant differences between the groups at the measurement time points are indicated by an asterisk (*).

A 40% increase in HR compared to when at rest, and thus a minimal workload, was achieved in all cases. Detailed HR values of the pugs and the CG at rest, during exercise, and during the recovery period are shown in Figure 1. The HRs of the groups were statistically significantly different at most time points (Figure 1). The Ts of the dogs are shown in Figure 2. Before exercise, the T of all dogs was within a normal range [41]. Increases in T occurred in both groups during exercise. The mean (± standard deviation (SD)) increase in pugs (0.5 °C ± 0.3 °C) was statistically significantly higher compared to the CG (0.1 °C ± 0.1 °C) (*p* = 0.0015). Pugs that reportedly suffered from heat intolerance (*n* = 18) had a statistically significantly lower mean (± SD) increase in T (0.3 °C ± 0.2 °C) than the other pugs (0.5 °C ± 0.1 °C) (*p* = 0.0250).

During exercise, most of the dogs started panting. Directly after exercise, 76% of the pugs panted compared to 25% of the dogs in the CG. In both groups, there were dogs that panted at the beginning (17% of the pugs and 13% in the CG) and at the end of the FT (12% of the pugs and 13% in the CG).

Most dogs in both groups recovered within 15 min after the end of the FT. Detailed recovery times of the groups can be found in Table 7. In terms of HR, four pugs and one dog in the CG did not reach resting values with a tolerance of 10% within 15 min. All dogs returned to the normal range of T during recovery. For RR, there were dogs in both groups that were still panting after 15 min of recovery. Taking into account the recovery times of HR, T, and RR, 81% of the pugs and 75% of the CG recovered within 15 min. There was no statistically significant difference between the recovery times of the two groups relating to HR, T, or RR.

### 3.2. Respiratory Noises and Breathing Patterns

None of the CG dogs produced any unphysiological RNs at rest or during exercise. The RNs observed in the pugs are shown in Table 2. During exercise, more RNs were observed, and existing noises became more prominent. An RN at rest audible without a stethoscope was heard in 45% of the pugs, with the majority being mild and intermittent. After exercise, in 67% of the pugs, an RN was audible without a stethoscope. Most RNs were a typical snoring sound, and were therefore determined as stertor (at rest, 72%; after exercise, 81%). At rest, 5%, and after exercise, 2%, of RNs could be identified as stridor. Not all RNs in pugs were able to be classified based on their origin (at rest, 10%; after exercise, 10%). In terms of inspiratory effort, 58% (30/52) of the pugs and 50% (4/8) of the CG showed a mildly increased respiratory effort after exercise. A moderately increased respiratory effort was observed in two pugs after 11 min, and in four pugs at the end of the FT. One pug had a severely increased inspiratory effort after five minutes of exercise. For this dog, the FT was stopped at this point.

### 3.3. Functional BOAS Grading

Four of the eight pugs that did not run on the treadmill but were classified as grade 2 or higher on the basis of the findings at rest were also listed in the results of the BOAS grading. In one pug, the FT was stopped after four minutes due to an increase in HR higher than 220 bpm. This dog was also assigned to the BOAS+ group, because it had already been classified as grade 3 due to the findings at rest. In total, the modified BOAS grading according to Liu et al. [43] could be applied to 56 pugs. Detailed classification of these pugs after 5, 10, and 15 min of exercise can be found in Table 3.

### 3.4. Results of the Fitness Test in Relation to BOAS Grading

**Figure 3:**
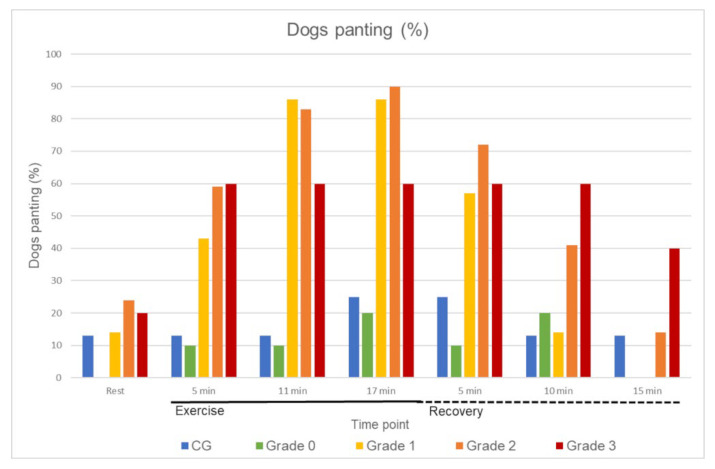
Percentage (%) of panting dogs in the control group (CG) and pugs divided into BOAS grades 0–3.

The baseline characteristics of all dogs included in the analysis can be found in Table 4. Age, body weight, and speed were not statistically significantly different between the CG and the BOAS groups (global hypothesis). HR (Table 5), T (Table 6), RR (Figure 3), and recovery (Table 7) were analysed considering the BOAS grading of the pugs.

In terms of HR, there was no statistically significant difference between the CG and BOAS grade 0 pugs at all measurement time points (pairwise comparison hypothesis, Table 5). BOAS grade 1–3 pugs had a significantly different HR compared to the CG at most time points (pairwise comparison hypothesis, Table 5). The T of the CG and pugs of BOAS grade 1 and 2 was statistically significantly different at the end of the FT (pairwise comparison hypothesis, Table 6). Temperature increase was statistically significantly different between the CG and BOAS grade 0–2 pugs (pairwise comparison hypothesis), whereby the dogs in the CG had the lowest mean (± SD) 0.1 °C (±0.1 °C) increase and the highest increase occurred in BOAS grade 1 0.54 °C (±0.32 °C) and grade 2 pugs 0.50 °C (±0.30 °C).

The percentage of panting dogs in the different groups can be found in Figure 3.

Statistically significant differences were found in recovery times of HR between dogs in the CG and BOAS grade 2 pugs (*p* = 0.0009, pairwise comparison hypothesis).

### 3.5. Body Conformation and Measurements and Their Impact on BOAS Grading

**Figure 4:**
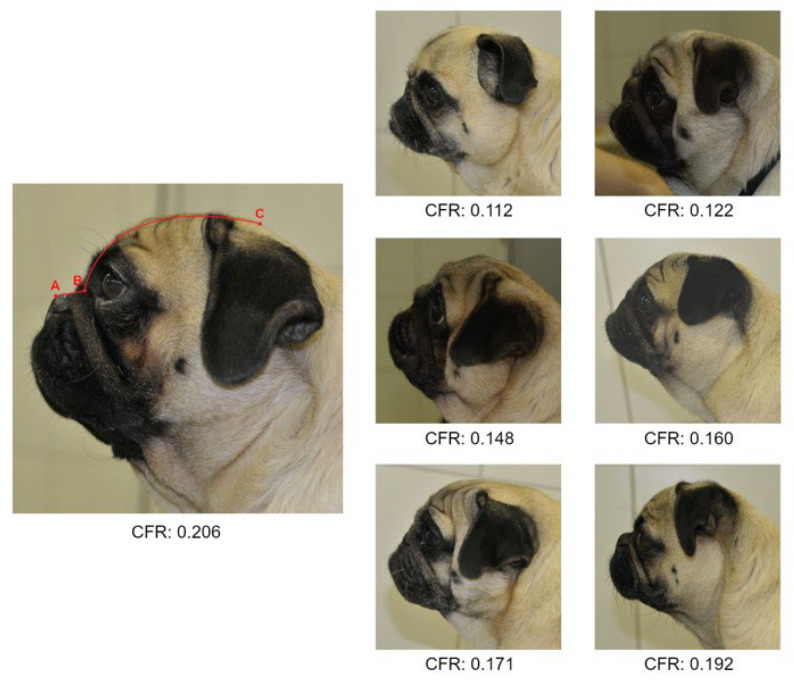
Examples of craniofacial ratios (CFR: ratio of muzzle length (A to B) to skull length (B to C)) measured in this study.

A total of 40% (25/62) of the pugs were overweight (BCS > 5/9). Fifty-two percent (32/62) of the pugs had at least moderate stenotic nares. The findings of the investigation concerning the influence of the variables on the BOAS grading assessed with univariable and multivariable logistic regression models are shown in Table 8. To demonstrate the appearance of the CFRs presented in this study, an overview of the sample images can be found in Figure 4. When testing the effect of the parameters using the univariate model, only the CFR was statistically significantly associated with the BOAS grading (*p* = 0.0114). Upon testing these influences via multivariable logistic regression, no factor had a statistically significant effect on BOAS grading. As neck girth and eye width were not measured in the first 13 pugs in this study, and BOAS grading could not be applied to every dog, only the measurements of 43 dogs could be included for calculations.

### 3.6. Owner Questionnaire

All participants in the study completed a questionnaire. A total of 62 questionnaires from pug owners and 10 from the CG were evaluated. Eighteen percent of pug owners and none of the CG stated that their dogs had a respiratory problem. Of the pugs, the age at which the problems occurred (mean ± SD, 3.3 ± 1.3 years) was only reported by six owners. The most common reason for not noting the age was that the dogs already showed respiratory problems at the time of purchase. Most pug owners reported that their dogs breathe quietly at rest (85%) and during exercise (65%). In the CG, 100% of the owners stated that their dogs breathe quietly in both situations. Regarding daily exercise capacity, 75% of the pug and 100% of the CG owners stated that their dogs were as resilient as other dogs. All pug owners said that their dogs had never had a choking fit in their life. Three of them (5%) reported that their dogs had shown cyanosis at least once in their life. Choking fits and signs of cyanosis were not observed by the owners in the CG. Forty-two (68%) of the pugs experienced reverse sneezing; thirty-seven (60%) of them rarely experienced reverse sneezing (once in a lifetime to a maximum of once per month) and five (8%) experienced it on a regular basis (about once per week). These results are comparable to those of the CG, where 70% of the owners reported that their dogs experienced reverse sneezing; 50% of them rarely experienced reverse sneezing (once in a lifetime to a maximum of once per month) and 20% experienced in on a regular basis (about once per week).

Regarding sleep, 98% of the pug owners and 100% of dog owners in the CG stated that their dogs slept calmly and deeply. Breathing pauses during sleep were reported by six pug owners (10 %) and choking fits during sleep by one owner (2%); neither of these symptoms were observed by any owner in the CG.

An overview of problems with eating observed by pug owners and owners in the CG can be found in Table 9.

A total of 58% of the pug owners stated that their dogs experienced no restrictions in everyday life. When asked about the restrictions (multiple answers possible), 18 owners (29%) reported restrictions because of heat intolerance, and nine (18%) because of breathing problems. Two (3%) responded that their dogs were restricted because of exercise intolerance, and one (2%) because of problems with feeding. None of the owners in the CG stated that their dog had any restrictions in everyday life. Most pug owners (95%) were aware prior to purchasing their dogs that pugs could have breathing problems. The last question, whether they would consider owning a brachycephalic dog again, was answered “yes” by most pug owners (87%).

## 4. Discussion

The primary objective of this study was to evaluate the applicability and utility of a treadmill-based submaximal FT in pugs. This included testing the feasibility of the specific exercise protocol under standardised conditions, and recording the physiological responses to the defined workload. Examination of physiological responses under standardised conditions was shown to be necessary, since reactions depend on the type, duration, and intensity of exercise [37,38,40,46,47]. Most of the dogs were willing to run on the treadmill after a short familiarisation period, even if they had never run on a treadmill before. By performing the FT on a treadmill, it was possible to observe the dogs during the entire testing period, to set a constant speed to ensure an uninterrupted, steady workload, and to monitor their HR continuously.

Due to possible restrictions in respiration and thermoregulation present in pugs, it was important that the workload remained in a submaximal range to avoid endangering the participants. This could be ensured through the continuous monitoring of their HR, and by stopping the FT if HR exceeded 220 bpm. Studies have shown that submaximal exercise tests are suitable for assessing daily exercise capacity in humans, and may be more closely related to daily activities than maximal exercise tests [28,29]. In addition, the mean (± SD) distance of 1.12 (± 0.1) kilometres was comparable to the 1000-metre walking test used in a previous study, which showed high sensitivity and specificity in identifying BOAS-affected pugs [11]. Within a range of four to eight kilometres per hour, an individual comfort speed could be set for each dog. Since the assumption of a sufficient workload was also supported by a 40% increase in HR in relation to values at rest, the individual comfort speed seemed appropriate to provide the desired workload.

The modified BOAS functional grading system used in the present study was developed by Liu et al. in 2015, using a whole-body barometric plethysmograph [43]. Since then, it has been applied in several studies on brachycephalic dogs [9,11]. The application was easy to implement. Although this scoring system cannot be considered as a definitive diagnostic tool, it has been shown to reflect the severity of BOAS [11,48], and enables clinicians to differentiate between affected and unaffected patients. The results of the BOAS grading in this study, which are mainly based on evaluating RN and breathing patterns, showed that with each subsequent FT level (5, 10, and 15 min), more pugs were assigned to higher grades. Regarding the evaluation of BOAS grading at the end of the FT, another eight pugs fell into the affected BOAS+ group that had been initially assigned to BOAS− upon evaluation after five minutes. For these dogs, RNs became more prominent, and were audible without a stethoscope after 15 min of exercise. Thus, a longer period of exercise resulted in a more reliable identification of pugs suffering from BOAS than after five minutes. Regarding the severe clinical signs shown by the two pugs that were not able to complete the FT, it does not seem advisable to breed these dogs, and dog owners should be discouraged by their veterinarian to do so. If the grading system by Liu et al. (2015) [43] and the clinical recommendations associated with this grading system are applied to the results of the performed submaximal FT, all dogs rated as BOAS+, which means 70% of the pugs examined in the study, should not be used for breeding.

The HR values of the pugs and CG were within the normal range at rest and after recovery [49]; however, the HRs of the two groups were statistically significantly different at most measurement time points, even at rest. It is known that HR can be influenced by training [50], and that fit dogs have a lower HR during exercise [51]. As half of the CG owners reported that they actively exercised their dogs (two owners reported doing agility regularly, and three dogs were used for hunting), this could be an explanation for the differences between the CG and pugs. It was also shown that HR varies in healthy dogs of different breeds, which may have also influenced the results [52,53]. Upon closer examination, however, when the pugs were further subdivided into the BOAS groups, the HRs of the grade 1–3 groups statistically significantly differed at most time points from the CG. This could indicate that as soon as pugs show any symptoms of BOAS, their fitness may be affected. Since the workload of the CG and the group of pugs, regardless of their BOAS subdivision, was comparable, a difference due to varying levels of strain was unlikely.

In terms of T values, the pugs had a statistically significantly higher T increase than the CG, which is in line with the findings of previous studies [11,48]. In dogs, heat loss is mainly achieved through the respiratory tract [54,55], whereby heat exchange via the nose plays an important role [56], and shortening of the nose results in restricted functionality [57]. T values of the pugs that reportedly suffered from heat intolerance were not conspicuous. They even had an unexpectedly lower T increase compared to the other pugs. All pugs and dogs from the CG showed T values within the normal range at rest and after 15 min of recovery [41]. It therefore seemed useful to record T in order to exclude overheating before the FT, especially in dogs with limited thermoregulation. However, based on these values, it was not possible to identify pugs suffering from reported heat intolerance. When comparing T between pugs subdivided into BOAS grades, BOAS grades 1 and 2 had statistically significantly higher T values at the end of the FT compared to the CG. The T increase in these groups was also higher compared to the CG. This may indicate that heat exchange is more compromised in dogs suffering from BOAS symptoms.

Regarding RR, there were proportionately fewer panting dogs in the CG compared to the pugs, and more panting dogs in grades 1 to 3 than in CG and grade 0 at all time points (Figure 3). Previous studies detected several reasons for dogs to pant: for heat exchange [58], as a response to stress [59], or as a possible indication of an upper airway obstruction [60]. Almost all dogs presented in this study were unfamiliar with their surroundings, and probably experienced a certain amount of stress when being admitted to the clinic. In the current study, one dog in the CG and nine pugs already panted before the FT and after the 15-min recovery period. When examining other respiratory symptoms of the dogs that panted continuously, the dog in the CG did not show any abnormalities. In the nine pugs, however, it was noticeable that most of them (*n* = 8) were graded as BOAS+ (grade 2 or 3) based on respiratory findings after 15 min of exercise. Accordingly, these dogs showed respiratory abnormalities in addition to panting. Therefore, due to the various causes of panting, it is important to additionally consider the breathing pattern, and pay attention to possible RN in order to check for a possible functional impairment, especially in pugs.

One requirement for passing the fitness test currently used in Germany in some kennels for acquiring breeding approval is that the dogs return to their resting values within a 15-min recovery period [32]. Most of the dogs in the present study succeeded in recovering within 15 min, especially regarding the recovery time of RR; almost all dogs in the CG and all BOAS− pugs were able to recover within 15 min. If the recovery time had been reduced to 10 min, 52% (*n* = 27) of the pugs and 38% (*n* = 3) of the CG would not have been able to return to resting or normal values. Of these 27 pugs, some showed signs of altered breathing (of the 27 pugs, 22 were graded as BOAS+ after 15 min), but there were also dogs experiencing no breathing problems. Thus, a 15-min recovery period appears to be advisable.

RN and breathing pattern assessments could be performed well, especially in combination with the FT. During exercise, RNs were observed more frequently and existing noises became more prominent. Thus, RN could be better assessed in relation to exercise. This is in accordance with findings from other studies [11,31]. Most RN (72% at rest, 81% after exercise) could be identified as stertor, the typical snoring sound [12,15]. An RN identified as stridor was observed in 18% of the pugs at rest, and was audible in 9% of cases after exercise; thus, it cannot be excluded that stridor was masked by the stertor, which became louder during exercise. Stridor could be detected in very young dogs in one study, so auscultation and evaluation seem to be reasonable at a young age [61]. Results of auscultation also have been shown to have a high correlation with findings under anaesthesia [31]. Additionally, in the present study, the breathing patterns were examined and assessed, especially regarding the inspiratory effort. A mild increase in inspiratory effort is a physiological response to exercise [62], but may also be an indication of increased airway resistance [63]. Furthermore, in the grading performed by Liu et al. (2015) [43], dogs with a mildly increased respiratory effort after exercise were still classified as BOAS−, and thus as functionally unimpaired.

Classification of BOAS after five minutes was chosen with regard to the assessment of the influencing factors in order to ensure that the results were comparable. In the present study, the CFR had a statistically significant influence on the BOAS grading in the univariable model, which was not confirmed in the multivariable model. Since BOAS is assumed to have a multicausal aetiology [9,25], the application of a multivariable model seems to be particularly relevant, and should therefore be preferred when evaluating possible influencing factors [64]. There are several studies investigating the influence of morphological characteristics on the presence of BOAS, with varying results. An example of this discrepancy were findings on the influence of stenotic nostrils, which indicated a large impact in two studies [9,26] but no influence in another study [11]. Moreover, investigations of X-ray indices and nostril opening in combination with a performed FT in accordance with the guidelines of the German Pug Club (Deutscher Mopsclub e.V.) showed no differences between pugs that successfully passed the test and those that failed [32].

When examining body conformation in the present study, it was noticeable that more than half of the pugs had at least moderate stenotic nares, although open nostrils were described as a breeding aim of the FCI [65]. This figure is even lower compared to other studies [11,26]. Even though the nostril opening was not shown to be a statistically significant influencing factor in the present study, and taking into account the varying results of other studies, the nostrils still represent a possible resistance of the upper respiratory tract, which could have a large impact on breathing [63]. Since stenosis of the nostrils is one of the few possible constrictions that can be assessed very easily from outside, more attention should be given to this.

The CFR had no statistically significant influence on being BOAS+ in the multivariable model. Nevertheless, since extreme brachycephaly is a risk factor for numerous other diseases [6,8], a benefit of breeding dogs with longer muzzles seems obvious. Additionally, as the nose plays a major role in thermoregulation [1], and several owners stated in our study questionnaire that their dogs suffered from heat intolerance, breeding for a lengthening of the muzzle might improve heat exchange in brachycephalic dogs [56]. Discrepancies in the results of other studies, and also the findings in the present study, underline that the emergence of BOAS is probably not due to a specific trait, but rather an interaction of many factors. Therefore, it seems to be a reasonable approach to assess the fitness and breathing of the dogs rather than one specific anatomical trait.

The results of the owner questionnaire showed that health restrictions, such as impaired breathing and signs of disturbed food uptake, were observed more frequently in pugs compared to the CG, which is in line with the findings of various previous studies [11,19]. Twelve pug owners reported that their dogs experienced breathing problems. The mean age (± SD) (3.3 ± 1.3 years) at which the first symptoms appeared is comparable to the age observed in other studies [10,66]. Of these twelve pugs, all but one were graded as BOAS+, and thus were functionally impaired according to this grading [43]. Severe problems, such as cyanosis or choking fits, were reported by only a few owners compared to another study that was conducted on dogs presented for BOAS surgery [19].

Two pug owners stated that exercise intolerance was the most limiting factor of their dogs. In one of the two dogs, the FT had to be stopped after four minutes due to an HR of over 220 per minute. The other case showed abnormalities in breathing with a moderate RN and a moderately increased inspiratory effort after exercise. This pug also did not recover after a 15-min recovery period in terms of RR. Thus, in this study, the two dogs with a reported limitation of exercise capacity were unable to successfully perform the FT.

However, out of nine owners who reported that their dogs were most affected by breathing restrictions, three stated that their dogs did not experience any breathing problems. Perceiving restrictions but not identifying them as a problem was also described in another study [18].

## 5. Conclusions

In the present study, it has been shown that the treadmill-based FT was tolerated by most of the dogs and can be applied under standardised conditions. The benefits of exercise testing on the treadmill are the continuous workload and an improved ability to observe the dogs during the entire examination period. The individual running speed of four to eight kilometres per hour seemed suitable, and a recovery time of 15 min was appropriate. Since functionally impaired dogs (BOAS+) and dogs with abnormalities in the form of RN and moderately increased inspiratory effort, were able to complete the FT, additional parameters, such as breathing pattern and RN, should be considered to reliably classify the dogs. Investigating possible risk factors for BOAS did not reveal additional information in this study population. Moreover, there are other breed-specific diseases to consider that may limit breeding suitability in addition to the performance of the FT and BOAS classification.

## Figures and Tables

**Table 1:** Measurement time points for heart rate (beats per minute (bpm)), rectal body temperature (°C), and respiratory rate (breaths per minute (breaths min^−1^)). Time points at which measurements were performed are marked with an “X”.

Time Point (min:sec)/Parameter	At Rest	02:30	05:00	06:00	08:30	11:00	12:00	14:30	17:00
Heart rate (bpm)	X	X	X	X	X	X	X	X	X
Temperature (°C)	X		X			X			X
Respiratory rate (breaths min^−1^)	X		X			X			X

**Table 2:** Respiratory noises (RN) of pugs at rest and during exercise.

Time Point		At Rest	5 min	11 min	17 min
**Number of subjects (*n*)**		62	52	52	52
**No RN (%)**		37	25	27	17
**RN audible without stethoscope (%)**					
Intermittent	Mild	34	15	13	19
	Moderate	2	0	2	2
	Severe	0	0	0	0
Constant	Mild	6	31	31	19
	Moderate	3	2	11	27
	Severe	0	2	0	0
**RN audible only with stethoscope (%)**					
Intermittent	Mild	8	13	0	6
	Moderate	0	0	8	0
	Severe	0	0	0	0
Constant	Mild	8	10	8	8
	Moderate	2	2	0	2
	Severe	0	0	0	0

**Table 3:** Number of pugs (*n* = 56) classified as grade 0, 1, 2, or 3 after 5, 10, and 15 min of exercise.

Time Point/Grading	BOAS−		BOAS+	
	Grade 0	Grade 1	Grade 2	Grade 3
5 min	14	11	24	7
10 min	12	7	30	7
15 min	10	7	32	7

**Table 4:** Baseline characteristics of included dogs. Characteristics were reported for dogs in the control group (CG), all pugs, and pugs divided into the BOAS groups (grades 0–3).

Variable/Group	CG	All Pugs	Grade 0	Grade 1	Grade 2	Grade 3
Number (*n*)	10	62	10	7	32	7
Male (%)	50	46.77	70	28.57	50	28.57
Age (years, mean ± SD)	6.2 ± 2.6	4.8 ± 2.4	4.5 ± 1.5	4.1 ± 1.7	4.6 ± 2.1	4.9 ± 2.2
Weight (kg, mean ± SD)	8.8 ± 1.9	8.9 ± 1.3	9.5 ± 1.1	8.2 ± 0.9	9.1 ± 1.21	7.8 ± 1.4
Speed (km/h, mean ± SD)	5.3 ± 0.7	5.0 ± 0.4	5.1 ± 0.5	4.9 ± 0.5	5.0 ± 0.4	4.8 ± 0.6

**Table 5:** Heart rate (mean ± SD) of dogs in the control group (CG) and pugs subdivided into BOAS grades 0–3.

Groups/Time Point (min:sec)		CG (*n* = 8)	Grade 0 (*n* = 10)	Grade 1 (*n* = 7)	Grade 2 (*n* = 29)	Grade 3 (*n* = 7)
**At rest**		103 ± 7	102 ± 9	**116 ± 10**	**118 ± 8**	**120 ± 12**
Exercise	02:30	148 ± 15	153 ± 25	**182 ± 24**	**171 ± 16**	**169 ± 22**
	05:00	149 ± 12	154 ± 17	**179 ± 20**	**172 ± 17**	**179 ± 19**
	06:00	121 ± 16	123 ± 19	**165 ± 13**	**156 ± 27**	145 ± 14
	08:30	155 ± 12	164 ± 17	**178 ± 18**	**170 ± 17**	**179 ± 11**
	11:00	153 ± 14	162 ± 15	**178 ± 17**	**169 ± 18**	**181 ± 13**
	12:00	123 ± 20	121 ± 24	**156 ± 16**	**141 ± 19**	**152 ± 30**
	14:30	152 ± 6	162 ± 14	**179 ± 18**	**172 ± 18**	**174 ± 7**
	17:00	153 ± 6	169 ± 17	**179 ± 21**	**176 ± 18**	**178 ± 18**
Recovery	05:00	107 ± 15	102 ± 16	**131 ± 21**	**132 ± 16**	**134 ± 21**
	10:00	103 ± 29	97 ± 16	119 ± 17	**123 ± 19**	123 ± 29
	15:00	94 ± 18	92 ± 17	111 ± 17	**115 ± 17**	**118 ± 21**

Significant differences between the BOAS groups compared to the control group (CG) are highlighted in bold.

**Table 6:** Body temperature (°C) in the control group (CG) and pugs subdivided into BOAS grades 0–3.

Groups/Time Point (min:sec)		CG (*n* = 8)	Grade 0 (*n* = 10)	Grade 1 (*n* = 7)	Grade 2 (*n* = 29)	Grade 3 (*n* = 7)
**At rest**		38.5 ± 0.3	38.4 ± 0.4	38.5 ± 0.3	38.5 ± 0.3	38.4 ± 0.5
Exercise	05:00	38.5 ± 0.3	38.4 ± 0.4	38.8 ± 0.3	38.7 ± 0.5	38.6 ± 0.5
	11:00	38.6 ± 0.2	38.4 ± 0.5	38.9 ± 0.3	38.8 ± 0.4	38.7 ± 0.2
	17:00	38.6 ± 0.2	38.6 ± 0.4	**39.0 ± 0.3**	**39.0 ± 0.5**	38.9 ± 0.3
Recovery	05:00	38.6 ± 0.2	38.6 ± 0.3	38.9 ± 0.3	38.9 ± 0.4	38.9 ± 0.4
	10:00	38.6 ± 0.2	38.5 ± 0.3	38.7 ± 0.4	38.7 ± 0.3	38.7 ± 0.4
	15:00	38.5 ± 0.2	38.4 ± 0.2	38.7 ± 0.3	38.7 ± 0.3	38.6 ± 0.3

Significant differences between the BOAS groups compared to the control group (CG) are highlighted in bold.

**Table 7:** Number of pugs (total and divided into BOAS grades 0–3) and dogs in the control group (CG) that recovered in ≤5, ≤10, ≤15 min or >15 min in terms of heart rate (beats per minute (bpm)), body temperature (°C), and respiratory rate.

Recovery Time/Groups		CG (*n* = 8)	All Pugs (*n* = 52)	Grade 0 (*n* = 10)	Grade 1 (*n* = 7)	Grade 2 (*n* = 29)	Grade 3 (*n* = 5)
Heart rate (bpm)	≤5 min	5	21	9	4	6	2
	≤10 min	1	20	1	1	16	1
	≤15 min	1	7	0	1	4	2
	>15 min	1	4	0	1	3	0
Body temperature (°C)	≤5 min	8	45	10	6	23	5
	≤10 min	0	2	0	0	2	0
	≤15 min	0	5	0	1	4	0
	>15 min	0	0	0	0	0	0
Respiratory rate	≤5 min	5	15	5	2	5	2
	≤10 min	2	17	3	3	10	1
	≤15 min	0	14	2	2	10	0
	>15 min	1	6	0	0	4	2

**Table 8:** Results of univariable and multivariable logistic regression.

Risk Categories	BOAS+		BOAS−		Univariable Model				Multivariable Model			
					OR	95%-CI		*p*	OR	95%-CI		*p*
						Low	Up			Low	Up	
	*n*	%	*n*	%								
total	25	58.1	18	41.9	x	x	x	x	x	x	x	x
**Gender**												
female (ref)	11	55.0	9	45.0	1	x	x	x	1	x	x	x
male	14	60.9	9	39.1	1.27	0.38	4.29	0.697	1.49	0.08	29.33	0.786
neuter status												
intact (ref)	16	51.6	15	48.4	1	x	x	x	1	x	x	x
neutered	9	75.0	3	25.0	2.81	0.64	12.41	0.172	0.09	0.01	1.27	0.073
BCS												
overweight (ref)	14	70.0	6	30.0	1	x	x	x	1	x	x	x
normal weight	11	47.8	12	52.2	0.39	0.11	1.38	0.146	2.15	0.26	17.70	0.465
nostril												
stenosis (ref)	13	56.5	10	43.5	1	x	x	x	1	x	x	x
open	12	60.0	8	40.0	1.15	0.34	3.90	0.818	1.06	0.15	7.30	0.956
**Continuous variables**												
age (per 1 month increase)	x	x	x	x	0.98	0.96	1.01	0.256	1.00	0.96	1.05	0.980
body length (per 1 cm increase)	x	x	x	x	1.24	0.94	1.63	0.127	1.35	0.84	2.16	0.211
neck girth (per 1 cm increase)	x	x	x	x	0.80	0.60	1.06	0.124	0.81	0.41	1.59	0.519
eye width (per 1 cm increase)	x	x	x	x	0.36	0.06	2.09	0.255	0.35	0.01	19.30	0.597
chest girth (per 1 cm increase)	x	x	x	x	0.80	0.60	1.06	0.113	0.71	0.42	1.19	0.186
height (per 1 cm increase)	x	x	x	x	1.16	0.89	1.52	0.270	0.90	0.48	1.68	0.720
CFR (per 1% increase)	x	x	x	x	1.33	1.04	1.69	**0.023**	1.35	0.93	1.97	0.114

BCS, body condition score; CFR, craniofacial ratio; CI, confidence interval; OR, odds ratio. The included CFR was converted from a ratio to a percentage for easier interpretation of the results; the significance level was set at *p* < 0.05; statistically significant results are highlighted in bold; x = not specifiable; ref = reference.

**Table 9:** Overview of problems with eating observed by the pug owners (*n* = 62) and the control group (CG) dog owners (*n* = 10).

Signs/Frequency		Never	Rarely	Regularly	Daily	Constant	No Specification
Regurgitation	Pugs (%)	92	4.8	0	0	1.6	1.6
	CG (%)	100	0	0	0	0	0
Vomiting	Pugs (%)	83.9	12.9	1.6	0	0	1.6
	CG (%)	60	40	0	0	0	0
Choking on food, difficulties swallowing	Pugs (%)	61.3	32.3	3.9	1.6	0	0
	CG (%)	40	60	0	0	0	0
Dyspnoea during feeding	Pugs (%)	93.6	1.6	1.6	1.6	0	1.6
	CG (%)	90	10	0	0	0	0

## Data Availability

The data presented in this study are available on request from the corresponding author.

## Supplementary Materials

The following supporting information can be downloaded at: https://www.mdpi.com/article/10.3390/ani12121585/s1, File S1: Questionnaire; Table S1: Assessment of respiratory noises and breathing patterns; Table S2: Body measurements.
