# Supplementary material for: Evaluation of a Treadmill-Based Submaximal Fitness Test in Pugs, and Collecting Breed-Specific Information on Brachycephalic Obstructive Airway Syndrome
Source: Animals (Basel). 2022 Jun 19;12(12):1585. doi: 10.3390/ani12121585 (PMC9219451; doi:10.3390/ani12121585)
Supplement: Supplementary file 1 [file animals-12-01585-s001.zip › S1 Questionnaire.pdf]

Supplementary Materials

Questionnaire

Medical history

**Pre-existing conditions**	
**Medication**	
**Vaccination status**	
**De-worming**	
**Preceding operations**	

Are you breeding dogs or have you talked about breathing problems with your breeder before purchasing your dog?

Yes ☐ No ☐

If yes, what is your opinion as a breeder/ as an informed purchaser (multiple choices possible)?

☐ Problems may occur in this breed
☐ There are no breathing problems in this breed
☐ Dogs of my kennel do not have breathing problems
☐ Only dogs that are not of a good kennel have breathing problems
☐ Both parents of this patient do not have breathing problems
☐ Breathing problems have occurred in this particular kennel
☐ Only dogs from abroad present with breathing problems
☐ Other (please state):

Does your dog have respiratory problems? Yes ☐ **No** ☐

<u>If YES</u>

Diagnosis? ____________________________

Treatment?
Operation: ______________________________________ none ☐
Medication: ____ none ☐

At what age did you first notice the problems? ______ Years _______Months

What signs did you notice first? (please write out)

____

Over what period of time did the problems occur?

Suddenly	Over several days	Gradually over a few weeks	Gradually over several months	Gradually over more than one year

Do you think this condition is …

Fixed	Gets worse	Remains the same	Inconsistent (but always present)	inconsistent (partially free of problems)

Does your dog suffer from his breathing problem?
(1 = does not suffer, 10 = suffers severely)

1	2	3	4	5	6	7	8	9	10

What is your dog like? Use the choices mentioned below to describe the character of your dog (multiple choices possible)?

☐ relaxed
☐ lazy
☐ chipper
☐ active
☐ curious
☐ shy
☐ brave

☐ aggressive
☐ submissive
☐ calm
☐ skittish
☐ sensitive
☐ inconspicuous
☐ dominant

☐ cheerful
☐ social (regarding other dogs)
☐ social (regarding other humans)
☐ antisocial (regarding other dogs)
☐ antisocial (regarding other humans)
☐ well-behaved

What is your dog's favourite activity? _________________________

Is your dog making sounds like the following awake and at rest*?

☐ snoring ☐ grunting ☐ wheezing ☐ stertorous breathing ☐ none

How often do you notice these sounds?

Constantly	Often (> once daily)	Daily (1 x per day)	Regularly (1x per week)	Rarely (< 1x per month	Never

* at rest = in a relaxed mood, resting/laying down

What does your dog's breathing sound like under the following circumstances?

	Very quiet	Panting, but no snoring/ wheezing	Light/mild snoring/ wheezing	Some snoring/ wheezing	Frequent snoring/ wheezing	Continuous snoring/ wheezing
At rest						
Daily life activity						
During exercise, e.g. on a walk, at play						
Asleep						

How often does your dog show restrictions of breathing in the following situations?

	Never	Rarely	Monthly	Weekly	Daily	More than daily
At rest						
Daily life activity						
During exercise, e.g. on a walk, at play						
Asleep						

How many walks do you take and how long are they (average per day):

Number: ______________________ Duration: _________________

My dog is exercised in sports: Yes ☐ No ☐

Sport type: ______________________ Frequency: ______________/Week

My dog lives in a household with other animals: Yes ☐ No ☐

If yes: What kind of animals? How many? ____________________

	Strongly agree	Rather agree	Neither	Rather not agree	Do not agree	No specification
My dog pants during exercise.						
My dog likes to play with other dogs.						
When playing, my dog can keep up with the other dogs.						
When playing, my dog runs a lot.						
After playing, my dog needs a long rest.						
My dog likes to explore the surroundings on walks.						
My dog is just as resilient as other dogs.						

How long can you walk with your dog?

	<10 min	10-30 min	30-60 min	>60 min	No specification
In summer					
In winter					

How long does your dog need to recover after exercise?

	Up to 5 min	Up to 15 min	Up to 30 min	Up to 60 min	No specification
In summer					
In winter					

At what temperature do the signs start to worsen (exercise intolerance, problems breathing)? (Please tick)

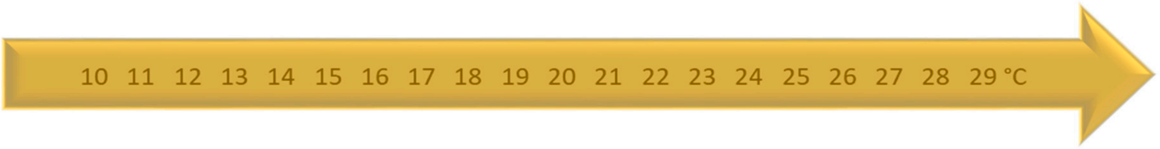

No worsening ☐ No specification ☐

	Strongly agree	Rather agree	Neither	Rather not agree	Do not agree	No specification
My dog does not like hot summer days.						
My dog prefers to rest rather than exercise on hot days.						
My dog pants more on hot days.						
My dog doesn't have a problem with heat.						
At home, my dog lies down right after a walk.						
My dog avoids the sun on hot days.						
I feel like my dog can't find rest on hot days.						
On warm summer days, my dog gets tired quickly.						

My dog is snoring:

Constantly	Often (once daily)	Daily (1 x per day)	Regularly (1x per week)	Rarely (< 1x per month)	Never

My dog presents with an increased respiratory effort*:

At rest:

Constantly	Often (once daily)	Daily (1 x per day)	Regularly (1x per week)	Rarely (< 1x per month)	Never

During/ after exercise:

Constantly	Often (once daily)	Daily (1 x per day)	Regularly (1x per week)	Rarely (< 1x per month)	Never

* for example nasal flaring, deep breaths or increasing breathing rate, marked movement/ pumping movement of the belly, abduction of the forelegs, stretching of the neck

Has your dog ever has passed out (= syncope)?

Constant	Often (> once daily)	Daily	Regular (once per month)	Occasionally (< once per month)	2 - 3 Times in its life	Once	Never

Has your dog ever suffered a choking fit?

Constant	Often (> once daily)	Daily	Regular (once per week)	Occasionally (< once per month)	2 - 3 Times in its life	Once	Never

Has your dog ever had a blue discoloration of his mucous membranes (= cyanosis, visible on gums and tongue)?

Constant	Often (> once daily)	Daily	Regular (once per week)	Occasionally (< once per month)	2 - 3 Times in its life	Once	Never

Has your dog ever shown "reverse sneezing"?

Constant	Often (> once daily)	Daily	Regular (once per month)	Occasionally (< once per month)	2 - 3 Times in its life	Once	Never

	Strongly agree	Rather agree	Neither	Rather not agree	Do not agree	No specification
My dog sleeps quietly and deeply.						
My dog can sleep badly to not at all.						
My dog can only sleep well when the chin is in an elevated position.						
My dog snores more in certain positions.						

Does your dog have breathing pauses during sleep (= sleep apnoea):

Constant	Often (> once daily)	Daily	Regular (once per month)	Occasionally (< once per month)	2 - 3 Times in its life	Once	Never	No specific ation

Does your dog have choking fits during sleep:

Constant	Often (> once daily)	Daily	Regular (once per month)	Occasionally (< once per month)	2 - 3 Times in its life	Once	Never	No specific ation

Food:

Type: (e.g. dry, wet, BARF, homemade diet):
____

Meals: ____/Day

How do you evaluate your dog's body condition?

- ☐ My dog is slightly overweight
- ☐ My dog is very overweight
- ☐ My dog has ideal weight
- ☐ My dog is slightly underweight
- ☐ My dog is very underweight

Has your dog ever suffered from the following problems during feeding? If yes, how often?

	Constantly	Daily	Regularly	Rarely	Never
Increased salivation					
Regurgitation*					
Vomiting**					
Choking on food/ difficulties swallowing					
Dyspnoea during feeding					

* regurgitation = mostly appearing directly after feeding, usually no signs to at most mild retching before regurgitation, food is discharged through the muzzle with seemingly little to no effort
** vomiting = usually announces itself before act, dog is apathic, uneasy or restless, trembling, salivating, smacking, dog retches strongly, abdominal wall is contracting visibly

My dog drinks

- ☐ little
- ☐ a normal amount
- ☐ a lot of water

These drinking habits

- ☐ are unchanged
- ☐ These drinking habits have recently changed
  If yes, since when and how? _______________

Urination:

- ☐ normal
- ☐ less than usual
- ☐ more than usual

Defecation:

- ☐ normal
- ☐ less than usual
- ☐ more than usual

Diarrhea: yes ☐ no ☐ occasionally ☐

In your opinion, what is the most restricting problem for your dog in everyday life?

- ☐ exercise intolerance
  What precisely?_____________
- ☐ breathing restrictions
- ☐ heat intolerance
- ☐ restrictions during feeding
- ☐ no restrictions
- ☐ no specification

Before acquiring the dog, did you know that breathing problems can occur in this breed?

Yes ☐ No ☐ Additional comments:_________________________________

Would you get a dog of a brachycephalic breed again?

Yes ☐ No ☐

Some questions were modified or compiled from the following publications:

Packer RMA, Hendricks A, Burn CC. Do dog owners perceive the clinical signs related to conformational inherited disorders as 'normal' for the breed? A potential constraint to improving canine welfare. Anim Welf. 2012;21(1):81-93.

Poncet CM, Dupre GP, Freiche VG, Estrada MM, Poubanne YA, Bouvy BM. Prevalence of gastrointestinal tract lesions in 73 brachycephalic dogs with upper respiratory syndrome. J Small Anim Pract. 2005;46(6):273-9.

Roedler FS, Pohl S, Oechtering GU. How does severe brachycephaly affect dog's lives? Results of a structured preoperative owner questionnaire. Vet J. 2013;198(3):606-10.
