# Supplementary material for: Evaluation of a Treadmill-Based Submaximal Fitness Test in Pugs, and Collecting Breed-Specific Information on Brachycephalic Obstructive Airway Syndrome
Source: Animals (Basel). 2022 Jun 19;12(12):1585. doi: 10.3390/ani12121585 (PMC9219451; doi:10.3390/ani12121585)
Supplement: Supplementary file 1 [file animals-12-01585-s001.zip › Tables S1 and S2.pdf]

Supplementary Materials

Table S1: Assessment of respiratory noises (RN) and breathing pattern in form of intensity of inspiratory effort and possible dyspnoea.

<table>
<tr><td>RN audible without stethoscope</td><td colspan="6">Yes</td><td colspan="6">No</td></tr>
<tr><td></td><td colspan="6">Intermittent</td><td colspan="6">Constant</td></tr>
<tr><td></td><td colspan="4">Mild</td><td colspan="4">Moderate</td><td colspan="4">Severe</td></tr>
<tr><td>RN audible with stethoscope</td><td colspan="6">Yes</td><td colspan="6">No</td></tr>
<tr><td></td><td colspan="6">Intermittent</td><td colspan="6">Constant</td></tr>
<tr><td></td><td colspan="4">Mild</td><td colspan="4">Moderate</td><td colspan="4">Severe</td></tr>
<tr><td>Association[A]</td><td colspan="4">Stertor (pharyngeal)</td><td colspan="4">Stridor (laryngeal)</td><td colspan="4">Not assignable</td></tr>
<tr><td>Inspiratory effort[B]</td><td colspan="3">Not present</td><td colspan="3">Mild</td><td colspan="3">Moderate</td><td colspan="3">Severe</td></tr>
<tr><td>Dyspnoea[C]</td><td colspan="3">Not present</td><td colspan="3">Mild</td><td colspan="3">Moderate</td><td colspan="3">Severe</td></tr>
</table>

[A] Association: Stertor: snoring sound; Stridor: harsh, high-pitched sound; not assignable: sound not identifiable as stridor or stertor.

[B] Inspiratory effort: mild: regular breathing cycle with minimal additional use of the diaphragm; moderate: distinct use of diaphragm and accessory respiratory muscles; severe: intensive use of the diaphragm and accessory respiratory muscles.

[C] Dyspnoea: mild: Signs of discomfort; moderate: irregular breathing; severe: irregular breathing with clear signs of discomfort.

Table S2: Measurements as described in Sutter et al. (2008).

**Measurement**	**Description**
Skull length	The distance from the occipital protuberance to the plane between the punctae lacrimale.
Muzzle length	The distance from the rostral end of the planum nasale to the plane between the punctae lacrimale.
Craniofacial ratio (CFR)	Ratio from muzzle length / skull length.
Eye width	The linear distance between the left and right punctae lacrimale.
Chest girth	The circumference of the deepest part of the thorax.
Neck girth	The circumference of the neck at the median distance between the external occipital protuberance and the withers.
Height	The linear distance from the ground to the cranial angle of the scapula.
Body length	The distance along the body from the dorsal plane of the withers to the point where the tail meets the body.
